# Association of Leukocytosis with Amphetamine and Cocaine Use

**DOI:** 10.1155/2014/207651

**Published:** 2014-01-22

**Authors:** John R. Richards, Valeria F. Farias, Chris S. Clingan

**Affiliations:** Department of Emergency Medicine, PSSB 2100, U.C. Davis Medical Center, 2315 Stockton Boulevard, Sacramento, CA 95817, USA

## Abstract

*Objective*. Determining the etiology of unexplained leukocytosis in asymptomatic patients may incur unnecessary testing, cost, and prolonged emergency department stay. The objective was to delineate if use of amphetamines and/or cocaine is a factor. *Methods*. For two years we reviewed all psychiatric patients presenting for medical clearance with exclusions for infection, epilepsy, trauma, or other nonpsychiatric medical conditions. *Results*. With a total of 1,206 patients, 877 (72.7%) amphetamines/cocaine-negative drug screen controls had mean WBC 8.4 ± 2.6 × 10^3^/*µ*L. The 240 (19.9%) amphetamines-positive, cocaine-negative, patients had WBC 9.4 ± 3.3 × 10^3^/*µ*L (*P* < 0.0001). The 72 (6.0%) amphetamines-negative, cocaine-positive, patients had WBC 7.1 ± 1.8 × 10^3^/*µ*L (*P* < 0.0001). The remaining 17 (1.4%) amphetamines/cocaine-positive patients had WBC 10.0 ± 4.2 × 10^3^/*µ*L (*P* = 0.01). Amphetamines-positive patients had a supranormal WBC ratio significantly higher than controls (23.8% versus 14.8%, *P* = 0.001), whereas only one cocaine-positive patient had a supranormal WBC count, with significantly lower ratio (1.4%, *P* = 0.0003). *Conclusion*. Use of amphetamines, not cocaine, may be associated with idiopathic leukocytosis. This may be explained by unique pharmacologic, neuroendocrine, and immunomodulatory differences.

## 1. Introduction

Patients who are acute or chronic stimulant users frequently present to the emergency department (ED) for myriad reasons [1]. Many have concomitant psychiatric disorders such as schizophrenia, bipolar disorder, and personality disorders which may shroud their illicit drug use [2]. These patients may be depressed and also present to the ED for suicidal or homicidal ideations and/or attempts [3]. Other ED patients may also be taking prescribed amphetamine compounds, such as methylphenidate (Ritalin) and amphetamine salts (Adderall) for conditions such as attention deficit hyperactivity disorder (ADHD) and narcolepsy [4]. Emergency physicians are frequently forced to provide “medical clearance” to these patients prior to their disposition [5]. These workups may be brief or lengthy depending on the institutional policy and treating clinician and may involve extensive lab testing and even radiographic studies, such as computed tomography of the brain. This represents questionable utilization of ED and hospital resources for the vast majority of these patients while contributing to the serious problem of ED crowding and hallway care [6].

Occasionally lab testing in this patient population may result in unexpected abnormal values, such as an elevated white blood cell (WBC) count in an asymptomatic patient. Disposition may be delayed until further testing is performed to investigate a possible infectious or inflammatory etiology, or a valid explanation is provided for the result (Table 1). This further delays patient transit through the ED to definitive care, worsens ED crowding, and increases cost. At our institution we have noticed patients with positive amphetamine drug screens will often have unexplained leukocytosis. To our knowledge, only one other study to date has also addressed this finding [7]. We conducted a retrospective review to further delineate this putative association between stimulants and leukocytosis and determine possible pharmacologic, neuroendocrine, and immunologic reasons to explain this phenomenon.

## 2. Methods

This study was a two-year retrospective review from January 2009 to December 2011. The setting was a university hospital ED with an associated emergency medicine residency program and Level I trauma designation. This ED has an average annual volume of 70,000 patients and serves an urban population of 2 million. It has a dedicated staff of psychiatric social workers 24 hours a day. As such, patients are often brought by prehospital and law enforcement personnel for medical clearance prior to disposition. This may include eventual placement in psychiatric or substance abuse treatment facilities or jail. This process is often lengthy, with some patients staying in the ED for days awaiting disposition after medical clearance. Many are placed on 72-hour involuntary “5150” holds. Medical clearance for these patients involves a standard set of lab tests, including complete blood count, chemistry panel, blood alcohol level, urine drug screen (UDS), acetaminophen, and salicylate levels. The UDS is qualitative but comprehensive, and includes cocaine, amphetamines (including methamphetamine and other derivatives), opioids, barbiturates, and benzodiazepines. Chart review was performed for the aforementioned time period, and all psychiatric patients were screened. Patients with elevated temperature were excluded, as were those with traumatic injuries, pregnant, epilepsy, or below the age of 18. This study was approved by our Institutional Review Board. Data was analyzed with Student's *t*-test, *χ* ^2^, and Fisher's exact tests with statistical significance assumed at a level of *P* < 0.05.

## 3. Results

Data from 1,206 patient visits were obtained and is summarized in Table 2. There were 485 (40.2%) females and 721 (59.8%) males. Average (± standard deviation) age and WBC count for the entire study group was 38.6 ± 13.4 years and 8.5 ± 2.8 × 10^3^/*μ*L, respectively. Median WBC was 8.1 × 10^3^/*μ*L. Normal range for WBC at our institution's laboratory is 4.5–11.0 × 10^3^/*μ*L. For the control subgroup, 877 patients had UDS negative for both amphetamines and cocaine. The average age of this control patient population was 38.7 ± 14.0 years, with 350 females (39.9%), 527 males (60.1%), and average WBC count 8.4 ± 2.6 × 10^3^/*μ*L. Median WBC count was 8.0 × 10^3^/*μ*L, and 130 patients (14.8%) had a supranormal WBC count (range 11.1–21.8 × 10^3^/*μ*L).

### 3.1. Amphetamine-Positive UDS

There were 240 patients with UDS positive for amphetamines and negative for cocaine. Average age was 36.6 ± 10.9 years, with 113 females (47.1%), 127 males (52.9%), and average WBC count 9.4 ± 3.3 × 10^3^/*μ*L. There was a statistically higher ratio of females to males in this subgroup compared to the controls (*χ* ^2^, *P* = 0.05), as well as lower age (*t*-test, *P* = 0.03). The average WBC count in this subgroup was significantly higher compared to the control group (*t*-test, *P* < 0.0001). Median WBC count was 8.9 × 10^3^/*μ*L, and 57 patients (23.8%) had a supranormal WBC count (range 11.1–28.4 × 10^3^/*μ*L). This supranormal WBC ratio was significantly higher than the control subgroup (*χ* ^2^, *P* = 0.001).

### 3.2. Cocaine-Positive UDS

There were 72 patients with UDS negative for amphetamines and positive for cocaine. Average age was 44.6 ± 11.8 years, with 16 females (22.2%), 56 males (77.8%), and average WBC count 7.1 ± 1.8 × 10^3^/*μ*L. There was a statistically lower ratio of females to males in this subgroup compared to the controls (*χ*², *P* = 0.003), as well as higher age (*t*-test, *P* = 0.0005). The WBC count in this group was significantly lower compared to negative controls (*t*-test, *P* < 0.0001). Median WBC count was 6.8 × 10^3^/*μ*L, and only one patient (1.4%) had a supranormal WBC count (11.1 × 10^3^/*μ*L). This supranormal WBC ratio was significantly lower than the control subgroup (Fisher's exact test, *P* = 0.0003).

### 3.3. Amphetamine and Cocaine-Positive UDS

There were 17 patients with UDS positive for both amphetamines and cocaine. Average age was 37.1 ± 11.9 years, with 6 females (35.3%), 11 males (64.7%), and average WBC count 10.0 ± 4.2 × 10^3^/*μ*L. The WBC count in this group was significantly higher compared to negative controls (*t*-test, *P* = 0.01). Median WBC count was 8.9 × 10^3^/*μ*L, and 5 patients (29.4%) had a supranormal WBC count (range 12.5–21.8 × 10^3^/*μ*L).

## 4. Discussion

The mechanisms behind leukocyte trafficking are complex, but essential, for an appropriate and effective immune response [8]. Antigenic and inflammatory stimuli, as well as psychological and physical stressors, are involved in the process of leukocytosis [9]. The regulation of the hematopoietic system is achieved at the cellular level of bone marrow stroma, at the humoral level by cytokines, catecholamines, neuropeptides, and hormones which interact with different immune cells [9]. As such, stimulant drugs, which affect central, autonomic, and peripheral nervous systems as well as the endocrine system, will affect leukocytosis [10, 11].

The physiological stress response is characterized by activation of the sympathetic nervous system and the hypothalamic-pituitary-adrenocortical (HPA) axis. This results in increased plasma concentrations of stress hormones such as catecholamines and glucocorticoids. This is accompanied by increases in circulating numbers of granulocytes, monocytes, and natural killer cells [12]. The number of T- and B-lymphocytes increases during acute stress but decreases during prolonged periods of stress [13, 14]. The role of dopaminergic neurons and modulation of immune response has been studied within the context of naturally occurring disease states. In patients with low levels of central nervous system (CNS) dopamine such as Parkinson's disease, there is a significant reduction in the proliferative response of peripheral lymphocytes, natural killer cell activity, and antibody production to various stressors [15, 16]. Conversely, in states of putative dopamine excess such as schizophrenia with mania, the majority of studies report an overall increase in the number of T-lymphocytes and interleukin concentration [17, 18]. Interestingly, treatment of schizophrenic patients with dopamine antagonist neuroleptics may actually have immunosuppressive effects [19].

Since dopamine cannot cross the blood-brain barrier, the specific correlation between CNS dopamine and the immune system is still under investigation. However, central dopamine levels appear to be a major influence the synthesis and release of other immunomodulatory neurochemicals. Peripheral dopamine also appears to be an important component of immunomodulation. Dopamine has been shown to be critical for trafficking and extravasation of T-lymphocytes across the blood vessels and tissue barriers [20, 21]. Peripheral dopamine also appears to modulate the functions of natural killer cells, splenic cells, macrophages, B-lymphocytes, and microglial cells. Presence of dopamine receptors and an active dopamine uptake and vesicular storage system in neutrophils, lymphocytes, and bone marrow cells have been demonstrated [22–25]. The synthesis and release of dopamine by lymphocytes suggest an intrinsic autocrine regulatory mechanism and, if from nonimmune tissues, a paracrine mechanism; stimulation of T- and B-lymphocyte proliferation in mice following pharmacological doses of dopamine has been established [26]. Tsao and coworkers reported injection of synthetic dopamine receptor-specific agonists enhanced lymphocyte proliferation [27]. This study group confirmed the effect when they also showed that the dopamine receptor antagonist, haloperidol, inhibited cell growth [28].

Amphetamines are potent sympathomimetic drugs, increasing synaptic levels of dopamine, norepinephrine, and serotonin within the synapse through multiple mechanisms [29, 30]. Although amphetamines bind to all monoamine transporters, its behavioral stimulant effects are mediated primarily through dopamine. Amphetamines disrupt vesicular storage of dopamine, thus allowing it to increase in the cytoplasm; these drugs also inhibit the degradative enzymes monoamine oxidases A and B. Amphetamines block the ability of the dopamine transporter to clear dopamine from the synapse and facilitate release of cytoplasmic dopamine across the cell membrane into the synaptic space. To our knowledge, the association of amphetamines and leukocytosis has been described in only one previous publication from 1983 [7]. Goldberg studied 13 patients presenting to the ED with amphetamine use. No patient had evidence of infectious or inflammatory disease that might cause leukocytosis. These patients were compared to a nonamphetamine cohort and found to have a statistically significant higher mean and median WBC count (12.9 × 10^3^/*μ*L and 8.7 × 10^3^/*μ*L versus 7.43 × 10^3^/*μ*L and 6.71 × 10^3^/*μ*L, resp.). Five of 13 patients (39%) had WBC count higher than normal. The author concluded it was important to recognize this putative association before embarking on time-consuming and expensive testing to determine the etiology of an amphetamine patient's unexplained leukocytosis.

Several animal studies support the association of leukocytosis and amphetamines. Llorente-García and coworkers administered amphetamine to rats and reported increased total peripheral leukocyte count but altered differential counts, with decreasing B-lymphocytes and increasing neutrophils and T-lymphocytes [31]. Glac et al. injected rats with amphetamine and reported differential effects on leukocyte populations in the peripheral blood, such as increased natural killer cells, granulocytes, and monocytes but decreased lymphocytes [32]. On average, the total number of WBC remained unaffected. Another potential mechanism involves the participation of microglial cells, which are involved in immune surveillance in the brain. Evidence suggests that activated microglial cells are triggered during the process of methamphetamine-induced toxicity in animals and this may result in peripheral immune system stimulation [33, 34].

Amphetamines also appear to influence leukocytosis through an indirect endocrine effect. Swerdlow and colleagues demonstrated amphetamine exposure and withdrawal in rats modify HPA axis endocrine responses, peripheral immune functions, and regional brain catecholamine levels [35]. The authors noted decreased adrenocorticotropin hormone (ACTH) levels, loss of the normal correlation between levels of plasma ACTH and corticosterone, and a significant increase in natural killer cell activity. Saito et al. injected monkeys with methamphetamine and noted serum cortisol concentration was significantly elevated nearly 3-fold and remained so for nearly 24 hours [36]. Natural killer cell and T-lymphocyte activity was significantly elevated at first but then dropped at 24 hours. They speculated these changes could be explained by the acute change in cortisone concentrations induced by methamphetamine. The phenomenon of glucocorticoid-induced leukocytosis has been described [37]. Mechanisms include enhanced release of polymorphonuclear leukocytes (PMNs) from bone marrow, delayed apoptosis of PMNs in the circulation, and reduced egress of PMNs into inflamed tissues, and demargination of PMNs from the intravascular pools. This is an acute effect, as WBC count will drop over time with chronic glucocorticoid presence.

Interleukins (IL) have also been shown to induce dermargination of PMNs as well [38]. House and associates evaluated the potential of synthetic and natural amphetamines to modulate cellular immune effector and regulatory mechanisms [39]. Exposure to amphetamine suppressed IL-2 production by T-lymphocytes, as well as a suppression of B-lymphocyte proliferation only at the highest amphetamine concentration examined. Natural killer cell function was slightly suppressed by amphetamine exposure but was enhanced by methamphetamine exposure. Exposure to naturally occurring cathinone resulted in stimulation of IL-2 production, B-lymphocyte proliferation, and cytotoxic T-lymphocyte activation. No significant effect of cathinone was noted on natural killer cell function. These data suggested natural and synthetic amphetamines exhibit differential and unpredicted immunomodulatory activity. Liu and colleagues demonstrated that exposure to methamphetamine increased the levels of the inflammatory cytokines IL-8, IL-1*β*, and TNF-*α* in human macrophages [40]. This finding raises the possibility methamphetamine may directly exacerbate inflammatory conditions that also modulate the immune signaling pathways.

In our study, there was a statistically significant lower WBC count and supranormal ratio in patients with only cocaine present in the UDS. Indeed, only one patient out of 72 had an elevated WBC count. The etiology of this finding is unclear, but there are some differences between amphetamines and cocaine that may be a factor. With regard to mechanism of action, cocaine has similarities but also important differences compared to amphetamines. Like amphetamines, cocaine binds to monoamine reuptake pumps, preventing removal of serotonin, dopamine, and norepinephrine from the synapse [41]. As with amphetamines, cocaine has been previously shown to activate the HPA axis, resulting in an increase in plasma ACTH, corticosterone, and beta-endorphin [42]. Unlike amphetamines, cocaine also acts as a muscarinic cholinergic antagonist and as a local anesthetic from sodium channel blockade [43]. Pellegrino et al. reported that the effects of cocaine did not appear to be due entirely to activation of the HPA axis and showed that peripheral administration of lidocaine and a synthetic monoamine reuptake inhibitor suppressed lymphocytosis [44]. They suggested both of these peripheral activities of cocaine may be involved in immunomodulation.

The half-life of cocaine is one hour, which is extremely short compared to amphetamines, which can range from 9 to 12 hours. This suggests the effect of cocaine on leukocytosis may be short-lived and not measurable at the time of these patients' presentation for medical clearance. The level of WBC was also significantly lower than the control group; perhaps the previously described glucocorticoid-induced leukocytosis may be more transient for cocaine than for amphetamines. Schmidt and colleagues showed that repeated exposure of rats to cocaine resulted in a long-lasting (weeks) sensitization of the HPA axis that was not seen with amphetamine exposure [45]. Cocaine has been shown to have an overall suppressive effect on the immune system [46, 47]. Amphetamine activation of certain pro-inflammatory cytokines, such as certain interleukins and TNF-*α*, has not been demonstrated for cocaine. Halpern et al. demonstrated that acute cocaine use resulted in suppression of proinflammatory IL-6 in human subjects [48]. Irwin and colleagues later showed decreased levels of proinflammatory IL-6 and TNF-*α* in both chronic and acute cocaine-exposed human subjects [49]. Mao and associates reported that cocaine downregulates IL-2-induced production of lymphocytes [50]. Cocaine has also been shown to increase levels of IL-10, which is an anti-inflammatory cytokine [51–53]. These stimulatory effects on the anti-inflammatory cytokines have not been shown for amphetamines and may explain the marked difference in leukocytosis between the amphetamine and cocaine subgroups.

## 5. Limitations

Certain psychiatric conditions such as untreated schizophrenia are associated with a hyperadrenergic state, and this may have affected measured WBC count. Psychiatric patients may have been taking medication that could theoretically alter their WBC count; we did not reconcile patients' medication lists, as most were noncompliant and unable/unwilling to provide this information during their ED stay. Similarly, we could not determine if the patient's stimulant use was acute or chronic, as this may have different effects on WBC count. We did not record the differential of each patient's WBC count to determine the predominant cell type responsible for the leukocytosis.

## 6. Conclusion

Based on these results, amphetamine, but not cocaine use, may be associated with a higher WBC count. This may be explained by the unique pharmacologic, neuroendocrine, and immunomodulatory differences between these drugs. Further studies involving healthy human subjects exposed to amphetamine and cocaine with consistent and structured methodology would be required to confirm this association. This would be important, as it may decrease the frequency of unnecessary testing to determine the etiology of leukocytosis in this asymptomatic ED patient population.

## Figures and Tables

**Table 1:** Causes of leukocytosis.

Infections
Bacterial
Fungal
Viral
Parasitic
Myeloproliferative disorders
Leukemia
Lymphoma
Polycythemia vera
Sickle cell disease
Inflammatory conditions
Connective tissue disease
Vasculitis
Myositis
Inflammatory bowel disease
Infarction (myocardial, pulmonary, etc.)
Burns
Hypersensitivity reactions
Metabolic disorders
Acidosis
Gout
Uremia
Eclampsia
Toxicologic
Endocrine (Stress leukocytosis)
Exercise
Stress
Excitement
Seizure
Drugs
Corticosteroids
Lithium
Heparin
Carbamazepine
Phenobarbital
Phenytoin
Minocycline
Clozapine
Catecholamines
Other
Neoplasm
Acute hemorrhage
Acute hemolysis
Postsplenectomy
Head trauma
Hypoxia
Idiopathic

**Table 2:** Differences between amphetamine and/or cocaine urine drug screen (UDS) positive and negative patients.

	Negative	Amphetamine	*P*	Cocaine	*P*	Amphetamine and cocaine	*P*
	UDS	positive UDS		positive UDS		positive UDS	
Total	877	240		72		17	
Female *n* (%)	350 (39.9%)	113 (47.1%)	0.05	16 (22.2%)	0.003	6 (35.3%)	0.8
Age ± SD	38.7 ± 14.0	36.6 ± 10.9	0.03	44.6 ± 11.8	0.0005	37.1 ± 11.9	0.6
Median WBC	8	8.9		6.8		8.9	
WBC ± SD	8.4 ± 2.6	9.4 ± 3.3	<0.0001	7.1 ± 1.8	<0.0001	10.0 ± 4.2	0.01
WBC > 11.0	130 (14.8%)	57 (23.8%)	0.001	1 (1.4%)	0.0003	5 (29.4%)	0.16

WBC units (×10^3^/*μ*L).
